# Novel Insights Into Sterol Uptake and Intracellular Cholesterol Trafficking During *Eimeria bovis* Macromeront Formation

**DOI:** 10.3389/fcimb.2022.809606

**Published:** 2022-02-11

**Authors:** Liliana M. R. Silva, Zahady D. Velásquez, Sara López-Osorio, Carlos Hermosilla, Anja Taubert

**Affiliations:** ^1^ Institute of Parasitology, Biomedical Research Center Seltersberg, Justus Liebig University Giessen, Giessen, Germany; ^2^ Veterinary Medicine School, CIBAV Investigation Group, University of Antioquia, Medellin, Colombia

**Keywords:** LDL sources, cholesterol trafficking, parasite acquisition of nutrients, ezetimibe, progesterone, U18666A

## Abstract

Apicomplexan parasites are considered as defective in cholesterol synthesis. Consequently, they need to scavenge cholesterol from the host cell by either enhancing the uptake of extracellular cholesterol sources or by upregulating host cellular *de-novo* biosynthesis. Given that *Eimeria bovis* macromeront formation in bovine lymphatic endothelial host cells *in vivo* is a highly cholesterol-demanding process, we here examined host parasite interactions based on host cellular uptake of different low-density lipoprotein (LDL) types, i.e., of non-modified (LDL), oxidized (oxLDL), and acetylated LDL (acLDL). Furthermore, the expression of lipoprotein-oxidized receptor 1 (LOX-1), which mediates acLDL and oxLDL internalization, was monitored throughout first merogony, *in vitro* and *ex vivo*. Moreover, the effects of inhibitors blocking exogenous sterol uptake or intracellular transport were studied during *E. bovis* macromeront formation *in vitro*. Hence, *E. bovis*-infected primary bovine umbilical vein endothelial cells (BUVEC) were treated with inhibitors of sterol uptake (ezetimibe, poly-C, poly-I, sucrose) and of intracellular sterol transport and release from endosomes (progesterone, U18666A). As a read-out system, the size and number of macromeronts as well as merozoite I production were estimated. Overall, the internalization of all LDL modifications (LDL, oxLDL, acLDL) was observed in *E. bovis*-infected BUVEC but to different extents. Supplementation with oxLDL and acLDL at lower concentrations (5 and 10 µg/ml, respectively) resulted in a slight increase of both macromeront numbers and size; however, at higher concentrations (25–50 µg/ml), merozoite I production was diminished. LOX-1 expression was enhanced in *E. bovis*-infected BUVEC, especially toward the end of merogony. As an interesting finding, ezetimibe treatments led to a highly significant blockage of macromeront development and merozoite I production confirming the relevance of sterol uptake for intracellular parasite development. Less prominent effects were induced by non-specific inhibition of LDL internalization *via* sucrose, poly-I, and poly-C. In addition, blockage of cholesterol transport *via* progesterone and U18666A treatments resulted in significant inhibition of parasite development. Overall, current data underline the relevance of exogenous sterol uptake and intracellular cholesterol transport for adequate *E. bovis* macromeront development, unfolding new perspectives for novel drug targets against *E. bovis*.

## Introduction

Apicomplexans are obligate intracellular protozoan parasites, which cause important diseases in humans and animals. In the bovine system, *Eimeria bovis* is one of the most pathogenic *Eimeria* species inducing cattle coccidiosis worldwide. The first round of asexual parasite replication (merogony I) is a highly energy- and building block-demanding process, especially in those *Eimeria* species showing macromeront formation [i.e., pathogenic species infecting cattle (*E. bovis*, *E. zuernii*), goats (*E. ninakohlyakimovae*, *E. aloingi*, *E. christenseni*), sheep (*E. bakuensis*), or camelids (*E. cameli* and *E. dromedary*)] that develop in endothelial cells of central lymphatic capillaries of the villi of the small intestine. Intracellular *E. bovis* macromeronts can reach up to 400 µm in size thereby inducing a substantial enlargement of infected host endothelial cells (Hammond et al., 1966). Of note, each macromeront produces up to 170,000 merozoites I, thereby being one of the most prolific apicomplexan parasites (Taubert et al., 2018). Therefore, in contrast to fast replicating coccidian parasites, such as *Toxoplasma gondii*, *Neospora caninum*, or *Besnoitia besnoiti*, macromeront-forming ruminant *Eimeria* species undergo a slow proliferative process over several weeks with a production of >1,000-fold offspring specimen (Taubert et al., 2018). Consequently, a high amount of cholesterol is required for offspring membrane synthesis and successful intracellular replication. However, coccidian parasites are considered auxotrophic for cholesterol (Bansal et al., 2005; Labaied et al., 2011; Coppens, 2013; Ehrenman et al., 2013; Hamid et al., 2014). Hence, successful replication of these parasites entirely relies on scavenging cholesterol from their host cells, either by enhancing host cellular endogenous *de-novo* synthesis or by upregulating low-density lipoprotein (LDL)-mediated cholesterol uptake from extracellular sources. Previous reports demonstrated that cholesterol acquisition occurs in a parasite-specific or even host cell type-specific manner. Hence, like *N. caninum* and *C. parvum* (Ehrenman et al., 2013; Nolan et al., 2015), *T. gondii* mainly scavenges cholesterol *via* enhanced LDL uptake but not *via* induction of *de-novo* synthesis in CHO cells (Coppens et al., 2000). Treatments with inhibitors of the mevalonate pathway (squalestatin) partially diminished *C. parvum* proliferation in infected intestinal epithelial cells, indicating an additional contribution of endogenous cholesterol synthesis in this process (Ehrenman et al., 2013). Interestingly, *Plasmodium* spp. hepatic stages accomplish cholesterol acquisition by both pathways, but they seem not essential for parasite replication (Labaied et al., 2011). In this context, few studies are currently available on slow developing coccidia, but these indicate distinct cholesterol acquisition pathways. Thus, an upregulation of molecules associated with both *de-novo* biosynthesis pathway and LDL-mediated cholesterol uptake was reported for *E. bovis*-infected host cells at the transcriptional level (Taubert et al., 2010; Hamid et al., 2014; Hamid et al., 2015). Likewise, treatments with inhibitors of host cellular cholesterol *de-novo* synthesis and esterification (e.g., lovastatin, zaragozic acid, CI976) significantly blocked parasite proliferation (Hamid et al., 2014). Moreover, an increased LDL receptor surface abundance was shown for *E. bovis*-infected endothelial host cells and excess LDL (10 µg/ml) supply boosted parasite proliferation (Hamid et al., 2015).

The aim of the current study was to examine host cellular uptake of different variations of LDL, i.e., of non-modified (LDL), oxidized (oxLDL), and acetylated LDL (acLDL) and their impact on *E. bovis* proliferation. Furthermore, the expression of lipoprotein-oxidized receptor 1 (LOX-1 or OLR1) mediating acLDL and oxLDL internalization was examined throughout *E. bovis* first merogony. Moreover, the effects of different inhibitors of exogenous sterol uptake or intracellular cholesterol trafficking on *E. bovis* macromeront formation were here studied.

## Materials and Methods

### Animals

Three parasite-free male Holstein Friesian calves obtained from a local dairy farm were treated with a single dose of 15 mg/kg bodyweight (bw) toltrazuril (Toltrazuril™ 50 mg/ml; Virbac, Vienna, Austria) and 0.2 mg/kg bw halofuginone (base as lactate salt) (Halocur^®^; MSD Animal Health, Kenilworth, NJ, USA) for seven consecutive days. Then, animals were kept in parasite-free conditions in autoclaved stainless steel metabolic cages (Woetho, Emmendingen, Germany) in a large animal stable (Institute of Parasitology, Justus Liebig University Giessen) equipped with a laminar flow lock entrance until experimental infection. Animals were screened for parasitic infections every 3 days. They were fed with milk substitute (Hemo Mischfutterwerk) and commercial concentrate (Raiffeisen). Water and sterilized hay were given *ad libitum*.

At the age of 7–8 weeks, calves were orally infected with 3.0 × 10^4^ *E. bovis* sporulated oocysts [these were washed thrice in water (600×*g*, 15 min) before infection]. Animals were monitored thoroughly during infection and blood samples were collected from the day of infection (0 d p.i.) until 21 d p.i. for diagnostic purposes.

### Parasites

*Eimeria bovis* strain H was initially isolated from the field in Germany and since then maintained by passages in parasite-free male Holstein Friesian calves (Fiege et al., 1992). Calves were orally infected, as described above, and subsequently, the collection, sporulation, and storage of oocysts were performed as previously described (Taubert et al., 2018). For excystation, oocysts were suspended in sterile 0.02 M L-cysteine HCl/0.2 M NaHCO_3_ solution and incubated for 20 h in a 100% CO_2_ atmosphere at 37°C. Then, oocysts were pelleted (600×*g*, 15 min, 20°C) and resuspended in 1× Hank’s balanced salt solution (HBSS; Gibco, Fisher Scientific GmbH, Schwerte, Germany) containing 0.04% (*w/v*) trypsin (Sigma-Aldrich, St. Louis, MO, USA) and 8% (*v/v*) sterile filtered (0.2 μm filter; Sarstedt, Nümbrecht, Germany) bovine bile obtained from the local abattoir. The oocysts were incubated for up to 4 h (37°C, 5% CO_2_) under constant microscopic control. Free sporozoites were washed twice (600×*g*, 15 min, 20°C) in medium (M199; Sigma-Aldrich), passed through a 10-μm pore-size filter (pluriStrainer, PluriSelect, Leipzig, Germany, Life Science) (López-Osorio et al., 2020), and counted in a Neubauer chamber.

### Host Cells and *Eimeria bovis* Infection Assays

Primary bovine umbilical vein endothelial cells (BUVEC) were isolated according to Taubert et al. (2018). Briefly, umbilical cords were collected under aseptic conditions from animals born by *sectio caesarea* and endothelial cells were isolated by treatments with 0.025% collagenase type II (Worthington Biochemical Corporation, Lakewood, NJ, USA) suspended in Pucks solution, which was infused into the lumen of ligated umbilical vein and incubated for 20 min (37°C, 5% CO_2_). The cell suspension was collected in cell culture medium (20 ml) and supplemented with 1 ml fetal calf serum (FCS, Gibco, Fisher Scientific GmbH, Schwerte, Germany). After washing (350×*g*, 12 min, 20°C), cells were resuspended in complete endothelial cell growth medium (ECGM, Promocell, Heidelberg, Germany, supplemented with 5% FCS), seeded in 25-cm^2^ tissue plastic culture flasks (Greiner, Frickenhausen, Germany), and kept at 37°C in 5% CO_2_ atmosphere. BUVEC were cultured in modified ECGM medium [EGCM diluted at 30% in M199 medium, supplemented with 5% FCS (Gibco), 1% penicillin and streptomycin (PS, *Sigma-Aldrich*)] with medium changes every 2–3 days. BUVEC layers were used for infection after one to two passages *in vitro*.

For the infection experiments, BUVEC layers (three or four biological replicates with four technical replicates, each), grown in 12-well plate formats (Falcon® Plates, Corning®, Wiesbaden, Germany), were infected at 80%–90% confluence with 7.8 × 10^4^ *E. bovis* sporozoites/well. The cell culture medium was changed 24 h after parasite infection and thereafter every 2–3 days.

### Lipoprotein Supplementation of *Eimeria bovis* Cultures

BUVEC (*n* = 3) were cultured on coverslips in modified ECGM supplemented with 1% PS and 5% FCS and infected with *E. bovis* sporozoites (2 × 10^4^ sporozoites per cm^2^ of host cell layer) as described above. To illustrate LDL, acLDL, and oxLDL uptake, labeled with Dil (1,1′-dioctadecyl-3,3,3′,3′-tetramethylindocarbocyanine perchlorate), *E. bovis*-infected (at 8, 14, and 17 d p.i.) and corresponding control BUVEC were either *i*) starved in basal medium (Promocell^®^) supplemented with 10% lipoprotein-deficient serum from fetal calves (LPDS; Sigma-Aldrich, S5394) for 24 h prior to the experiments or *ii*) continuously cultured in modified ECGM. Thereafter, Dil-LDL (10 µg/ml, Invitrogen, L3482), Dil-acLDL (10 µg/ml, Invitrogen, Waltham, MA, USA, L3484), or Dil-oxLDL (5 µg/ml, Invitrogen, L34358) was supplemented for 24 h (37°C, 5% CO_2_). BUVEC were washed thrice with PBS to remove any traces of labeling and fixed with 4% paraformaldehyde (PFA, 15 min, RT; Sigma-Aldrich). After three washes in PBS, samples were mounted in Fluoromount-G™ with DAPI (Invitrogen, 4959-52).

For exogenous LDL enrichment experiments, LDL (25 and 50 µg/ml final concentrations; Invitrogen, L3486), acLDL (10, 25, and 50 µg/ml final concentrations; Invitrogen, L35354), or oxLDL (5 µg/ml final concentration, concentrations above this were proven toxic for BUVEC; Invitrogen, L34357; human origin, tested in bovine pulmonary artery epithelium for recognition of scavenger receptors, oxidized by copper-mediated process) were supplemented to *E. bovis*-infected cell cultures from 10 d p.i. onwards. All the mentioned concentrations refer to the total mass of the particles. Therefore, LDL-supplemented medium was replaced every 2 days until the end of the experiment (24 d p.i.). To estimate the effects of supplementation on parasite proliferation, the number of meronts I per area (800 µm × 600 µm) and meronts I sizes (µm^2^) were evaluated at 15 and 19 d p.i. Moreover, *E. bovis* proliferation was determined *via* analysis of merozoite I production estimating *E. bovis* micromene protein 4 (EbMIC4-qPCR) at 24 d p.i.

### 3D Holotomography and Lipid Droplet Visualization

For live-cell 3D holotomography, BUVEC (*n* = 3) were seeded into 35-mm low-rimmed tissue culture µ-dishes (Ibidi^®^, Gräfelfing, Germany), grown overnight and infected with *E. bovis* sporozoites. At 4, 8, 12, 17, and 22 d p.i., holotomographic images were obtained by using 3D Cell Explorer microscope (Nanolive, Tolochenaz, Switzerland 3D-fluo) equipped with a ×60 magnification (*λ* = 520 nm, sample exposure 0.2 mW/mm^2^) and a depth of field of 30 µm. For lipid droplet visualization, BUVEC were stained with LipidSpot™ 488 (1:1,000, Biotium; 30 min, 37°C, in the dark). After incubation, live-cell 3D holotomography and analysis of LipidSpot™ 488-based fluorescence were performed in parallel and merged to prove lipid droplet identity (Silva et al., 2019). Images were analyzed using STEVE software (Nanolive, Tolochenaz, Switzerland) to obtain refractive index (RI)-based z-stacks (Sandoz et al., 2018).

Additionally, for confocal microscopic analysis of cellular lipid droplet distribution during the first merogony, BUVEC (*n* = 3) were grown on coverslips in modified ECGM supplemented with 1% PS and 5% FCS and infected with *E. bovis* sporozoites (2 × 10^4^ sporozoites/cm^2^ host cell layer) as described above. Infected cells were stained with LipidSpot™ 488 (1:1,000, Biotium, Fremont, CA, USA; 30 min, 37°C, in the dark) and fixed as previously described. After two washes in PBS, samples were mounted in Fluoromount-G™ with DAPI (Invitrogen, 4959-52).

### *Eimeria bovis* Merozoite I Quantification *via* qPCR

At 24 d p.i., supernatants of *E. bovis*-infected BUVEC were collected and cells were trypsinized and pelleted (900×*g*, 12 min) all together. Pellets were resuspended in 200 µl PBS, supplemented with 200 µl buffer AL (Qiagen, Hilden, Germany) and 20 μl proteinase K (20 mg/ml; Qiagen), and incubated at 56°C for 15 min (with agitation). DNA isolation was further performed according to the instructions of the manufacturer (DNeasy Blood & Tissue Kit, Qiagen), and samples were frozen at −20°C until further use. Real-time PCR for merozoite I quantification (Lutz, 2009) used EbMIC4 sequences according to (Hamid et al., 2014) and performed in a 20 μl total volume containing 0.8 μl (10 μM) EbMIC4 forward (5′→3′ CACAGAAAGCAAAAGACA) and reverse (5′→3′ GACCATTCTCCAAATTCC) primers, 0.4 μl (10 μM) probe (reporter 5′–3′ quencher: FAM/BHQ-1 CGCAGTCAGTCTTCTCCTTCC), 5 μl DNA, 3 μl H_2_O distilled, and 10 μl 2× PerfeCTa qPCR FastMix II (Quantabio, Beverly, MA, USA). The reaction conditions were as follows: 95°C for 10 min, 40 cycles at 95°C for 10 s, 60°C for 15 s, and 72°C for 30 s. PCRs were performed on a Rotor-Gene Q cycler (Qiagen, Hilden, Germany). The PCR data were extrapolated to standard curves using DNA from known numbers of merozoites I.

### LOX-1 Quantification in BUVEC and Serum Samples of *E. bovis*-Infected Calves

Protein levels of LOX-1 (syn. OLR1) in *E. bovis*-infected BUVEC and non-infected controls were quantified by a commercially available bovine lectin like oxidized low-density lipoprotein receptor 1 (LOX-1) ELISA kit (DL-Develop, Wuxi, China). BUVEC (*n* = 3) were grown in 75-cm^2^ cell culture flasks (Greiner) and infected with *E. bovis* sporozoites as described above. Cells were harvested at 8, 12, 15, and 20 d p.i., according to the instructions of the manufacturer. Non-infected cells were processed in parallel as controls. Cell layers were washed with PBS, then trypsinized and pelleted (400×*g*, 12 min). Cell pellets were washed three times with PBS and the number of cells per sample was determined before ultrasonication (3 times for 20 s on an ice bath). Samples were centrifuged at 1,000×*g* (15 min, 4°C) to remove cell debris and the supernatants were stored at −20°C until being processed *via* ELISA kit. Additionally, LOX-1 concentration was estimated in blood plasma samples of experimentally *E. bovis*-infected calves (*n* = 3) at days 0, 7, 14, 19, and 21 p.i. and analyzed *via* bovine LOX-1 ELISA kit (DL-Develop) according to the instruction of the manufacturer.

### Inhibition Experiments

For inhibitor treatments, *E. bovis*-infected and non-infected BUVEC (*n* = 3 or 4) were treated with pharmacological blockers from 10 d p.i. onwards. Hence, BUVEC were treated with ezetimibe (20 µM; Cayman Chemical, Ann Arbor, MI, USA), ezetimibe-glucuronide (30 µM; Santa Cruz Biotechnology, Inc., Heidelberg, Germany), polycytidylic acid potassium salt (poly-C; 100 µg/ml; Sigma-Aldrich, P4903), polyinosinic acid (poly-I; 100 µg/ml; Sigma-Aldrich, P4154), and sucrose (100 mM; Sigma-Aldrich, S7903) serving as inhibitors of sterol uptake. Additionally, progesterone (25–75 µM; Sigma-Aldrich, P0130) and U18666A (7.5 µM; Cayman Chemical Company, 10009085) were used, which are both known to block intracellular sterol transport and release from endosomes. As solvent control, either water, ethanol, or dimethyl sulfoxide was used.

### Viability Assays

Analysis on cytotoxic effects was performed for all inhibitors used in the current study *via* CyQUANT XTT^®^ Cell Viability Assays (Invitrogen; **Supplementary Figure 1**). Therefore, non-infected BUVEC (*n* = 3) were exposed for 72 h to varying inhibitor concentrations [poly-I (3.125–100 µg/ml), poly-C (6.25–200 µg/ml), ezetimibe (7.5–60 µM), sucrose (7.80–250 mM), U18666A (7.81–250 µM), progesterone (3.91–125 µM), ezetimibe-glucuronide (30 µM)] and processed according to the instructions of the manufacturer. Additionally, trypan blue exclusion assays (Sigma-Aldrich) were utilized to analyze the potential toxic effects of inhibitors (1 h treatment) on *E. bovis* sporozoite viability.

### Image Acquisition and Analyses

For fluorescence analyses, cells were analyzed using an inverted fluorescence microscope (IX81, Olympus, Shinjuku City, Tokyo, Japan) applying the UV filter set (340–380 nm excitation, 430 nm pass filter). Confocal images for *E. bovis*-infected BUVEC (17 d p.i.) for LDL, oxLDL, and acLDL detection were collected with a Zeiss Confocal LSM 710 equipped with a motorized XY stage (Objective Plan-Apochromate ×40, numerical aperture 1.3 Oil DIC MC27). Two channels were recorded (Blue/DAPI/405-laser and Red/HeNe-543 laser) for covering the whole macromeront size (1.07 µm slices z-stack). 3D reconstruction images for lipid droplets by live-cell imaging detection in *E. bovis*-infected BUVEC (15 and 22 d p.i.) were acquired with a ReScan Confocal Microscope instrumentation (RCM 1.1 Visible, Confocal.nl, Amsterdam, The Netherlands) equipped with a fixed 50-µm pinhole size and combined with a (Nikon, Düsseldorf, Germany) Ti2-A inverted microscope. The microscope was equipped with a motorized Z-stage (DI1500). The RCM unit was connected to Toptica CLE laser with the following excitations: 405/488/561/640 nm. Images were taken *via* a sCMOS camera (PCO edge) using a Plan Apo *λ* ×60 immersion oil objective (NA = 1.4; WD 130 µm; Nikon®). The setup was operated by the microscope software NIS-Elements (version 5.11). Image processing was carried out by ImageJ using merged channel plugins and restricting to minor adjustment of brightness and contrast as well as a 3D deconvolution module, NIS-Element module (Nikon®).

### Statistical Analyses

Statistical analyses were performed using GraphPad^®^ Prism (version 9.0.1 for Windows, GraphPad Software, San Diego, CA, USA, www.graphpad.com). Kruskal–Wallis tests were performed followed by Dunn’s multiple comparison test. If more than 20 observations were to be considered, normality was tested with the Shapiro–Wilk test. After one-way ANOVA or Kruskal–Wallis tests, Tukey’s multiple comparisons test or Dunn’s multiple comparison test was performed. When the percentage of control comparisons was considered, Dunn’s multiple comparison test followed by Kruskal–Wallis tests or Kolmogorov–Smirnov test was performed. *p*-values <0.05 were considered significant. Test groups were compared with the control group. Mean and standard deviation are depictured in the corresponding graphics.

## Results

### Live-Cell 3D Holotomographic Microscopy Illustrates High Demand and Storage of Lipids During *Eimeria bovis* Macromeront Development *via* Lipid Droplet Accumulation

**Figure 1:**
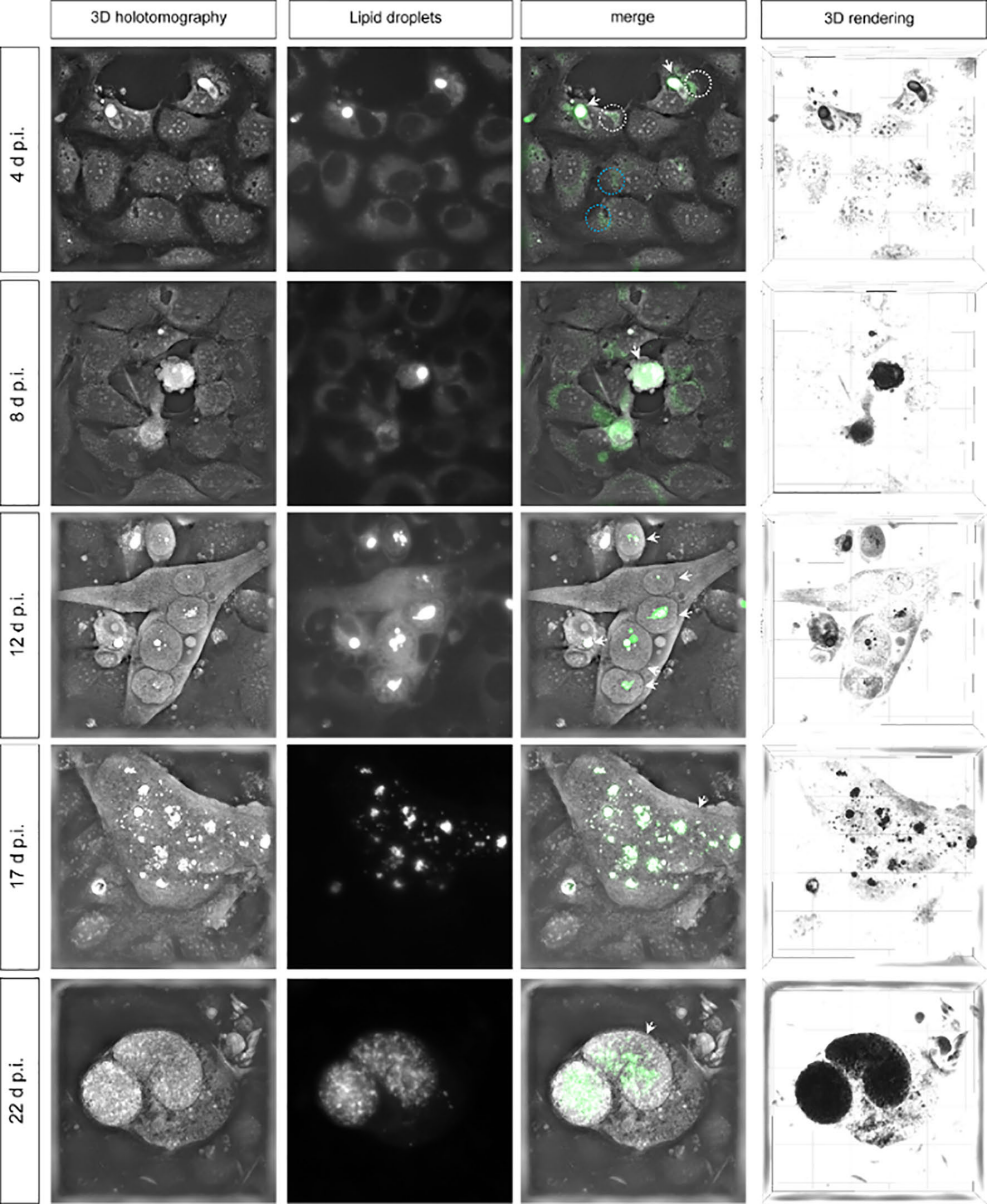
Lipid droplet formation in *Eimeria bovis*-infected host cells. Live-cell 3D analyses of *E. bovis*-infected host cells revealed the presence of an increasing number of lipid droplets during macromeront formation. 3D holotomography represents refractive index (RI) images; lipid droplets are stained by LipidSpot™ (green); merges show co-localization of white structures with higher RI and LipidSpot™-stained lipid droplets (green). 3D rendering shows lipid droplets in black in a 3D model. White arrows indicate *E. bovis* stages; ×600 images.

During first merogony, each *E. bovis* macromeront can produce >170,000 merozoites I thereby requiring a considerable amount of lipids for host cell membrane enlargement and offspring membrane production (Hamid et al., 2015; Taubert et al., 2018). Given that lipids are mainly stored in lipid droplets, the presence of these structures was studied *via* live-cell 3D holotomography and LipidSpot™-based epifluorescence throughout merogony I *in vitro* (**Figure 1**). As an interesting finding, we detected seemingly enlarged lipid droplets in the host cell cytoplasm soon after infection at day 4 p.i. (encircled in white, **Figure 1**) when considering the size of lipid droplets in non-infected control cells (encircled in blue). In addition, lipids in close association to refractile bodies of sporozoites were stained by LipidSpot™ (white arrows, **Figure 1**). In general, LipidSpot™-positive lipid droplet-like structures were detected in the cytoplasm of non-infected cells as well as in the cytoplasm of infected cells until 8 d p.i. (**Figure 1**). At 8 d p.i., the early meront typically rounds up leading to a more globular shape of infected host cell, thereby showing an intense LipidSpot™-based staining that hardly allows for the differentiation of host cell cytoplasmic and parasitic compartment. Interestingly, lipid droplet positioning within the meront differed with the state of maturation. Thus, from 12 d p.i. onwards, an enhanced number of eventually larger lipid droplets that were clustered into larger groups in a rather central position within the meronts were observed (**Figure 1**). From then onwards, both immature (17 d p.i.) and mature (22 d p.i.) macromeronts presented an accumulation and grouping of lipid droplets of different sizes but identical RI (as defined by 3D holotomography). The lipid droplet-based clustering effect and central lipid droplet positioning ceased when macromeronts fully matured, i.e., when merozoites I were formed. Thus, at 22 d p.i., numerous lipid droplets of smaller sizes were found more evenly distributed and seemingly associated with fully developed merozoites I (**Figure 1**). Technically, when using 3D holotomographic microscopy and merging RI-illustrated organelles with LipidSpot™-derived signals, these structures entirely overlapped thereby confirming their lipid droplet nature. However, epifluorescence-related staining revealed a lower resolution: while an accumulation of several single lipid droplets was apparent *via* 3D holotomography, larger and confluent structures were detected by epifluorescence (**Figure 1**: 12 and 17 d p.i.).

**Figure 2:**
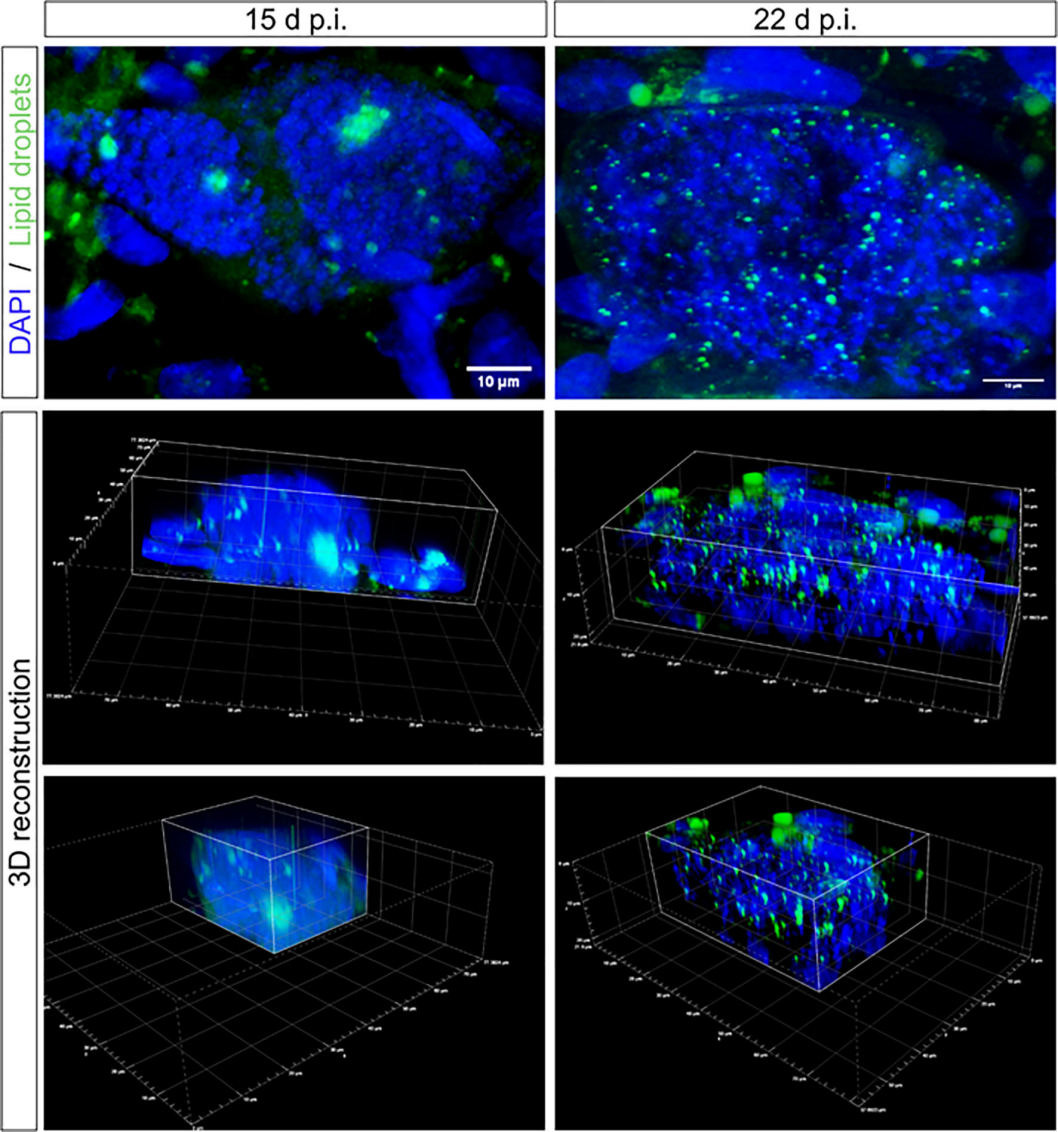
Lipid droplet distribution in *E. bovis* macromeronts. Confocal microscopy analysis evidenced the presence of large lipid droplets being positioned as large clusters in immature macromeronts at day 15 p.i. In contrast, in mature macromeronts (22 d p.i.), numerous small lipid droplets (green) were evenly distributed in the parasitic compartment as also illustrated by 3D reconstructions. DAPI (blue) was used to stain DNA. Scale bar 10 µm.

To confirm that lipid droplets were indeed located inside the meronts, confocal microscopic analysis was performed, evidencing a redistribution of lipid droplets from the host cellular cytoplasm (as observed in non-infected cells and until 8 d p.i.) into the meronts from 12 d p.i. onwards. Moreover, it was clearly visible that at 15 d p.i., larger and concentrated accumulations of lipids were located in more central positions within the meronts, while at 22 d p.i., lipid droplets appeared to disperse within the macromeront showing a smaller and more homogeneously spread lipid droplet phenotype (**Figure 2** and **Supplementary Videos 1**, **2**).

### Different LDL Modifications Are Internalized by *Eimeria bovis*-Infected BUVEC but Fail to Boost Parasite Replication

**Figure 3:**
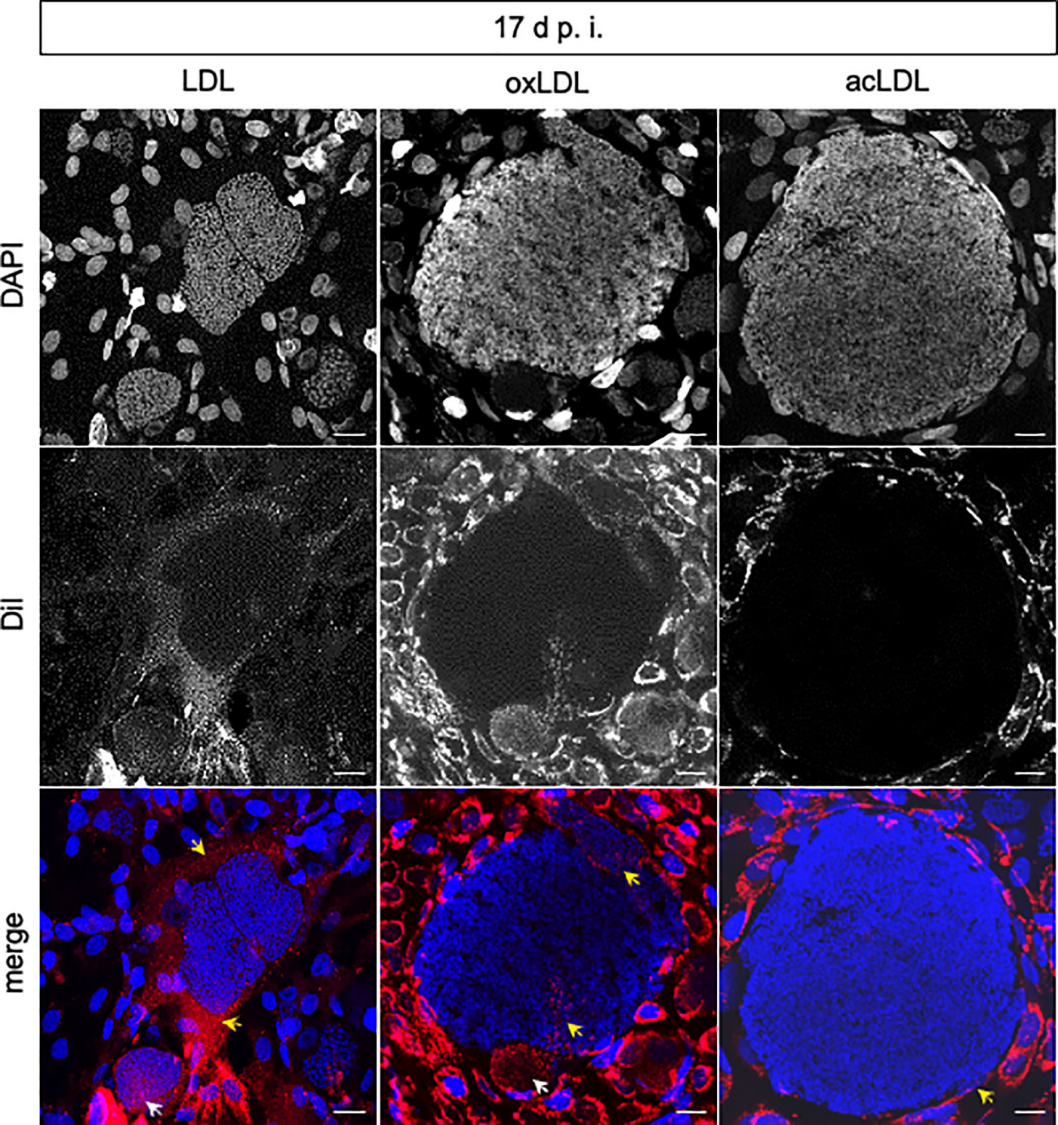
Internalization of LDL, acLDL, and oxLDL by *E. bovis*-infected host endothelial cells and meronts I. Confocal analyses revealed that Dil-LDL, Dil-oxLDL, and Dil-acLDL (red) were internalized into the host cell cytoplasm at all time points studied (*n* = 3, representative illustration). Intrameront LDL and oxLDL internalization (white arrows) was observed at 17 d p.i. In contrast, acLDL was not observed within meronts I. DAPI (blue) stained DNA. Internalization of marked lipids in the cytoplasm of host cells (LDL, oxLDL, acLDL) or stretches of the parasitophorous vacuole membrane (oxLDL) are signed with yellow arrows. Scale bar 20 µm.

To monitor if different LDL types are indeed internalized into *E. bovis*-infected cells or even into meronts, the uptake of fluorescent LDL, acLDL, and oxLDL was visualized in infected and non-infected BUVEC at days 8, 14, and 17 p.i. (**Figure 3**). Since Dil, the fluorescent compound used here as tracker is lipophilic, we assume that its presence in the different intracellular compartments directly relates to the presence of the associated LDL modification in each experiment. In contrast to our expectations, starved BUVEC internalized lower LDL proportions than non-starved cells. This phenomenon may be related to cell stress-related reactions in starved BUVEC. Consequently, only non-starved BUVEC were further analyzed (**Figure 3**). Overall, all LDL types (Dil-LDL, Dil-acLDL, and Dil-oxLDL) were in principle internalized by non-starved BUVEC and subsequently found in the BUVEC cytoplasm. In infected cells at 14 d p.i., exclusive signals of non-modified LDL were found and indeed were—to a minor degree—also apparent within the macromeront. Later, the different LDL types showed different uptake and distribution behavior in infected host cells. Thus, at 17 d p.i., non-modified LDL was equally internalized into both macromeront (**Figure 3**, white arrow) and host cell cytoplasm (**Figure 3**, yellow arrows). In contrast, oxLDL was only partially internalized and related signals were detected in both meront chambers (white arrow) and certain stretches of the parasitophorous vacuole membrane (**Figure 3**, oxLDL (yellow arrow), and **Supplementary Figure 2**). In contrast, acLDL was not at all detected in macromeronts, but exclusively occurred in the host cell cytoplasm (**Figure 3**, acLDL, and **Supplementary Figure 3**, z-stack analysis).

As an additional interesting finding, macromeront-carrying BUVEC were consistently observed to be closely surrounded by several non-infected host cells (also on the top and bottom of infected host cells) thereby seemingly enclosing the *E. bovis*-infected host cell. This finding resembled a kind of protective or supporting system for infected host cells being formed by non-parasitized bystander cells. In the current study, this phenomenon was observed at days 14 and 17 p.i., with the nuclei of the “supporter cells” appearing directed and aligned to the membrane of the infected host cell. These peculiar observations were rather evident in BUVEC carrying globular macromeronts than in flattened host cells.

**Figure 4:**
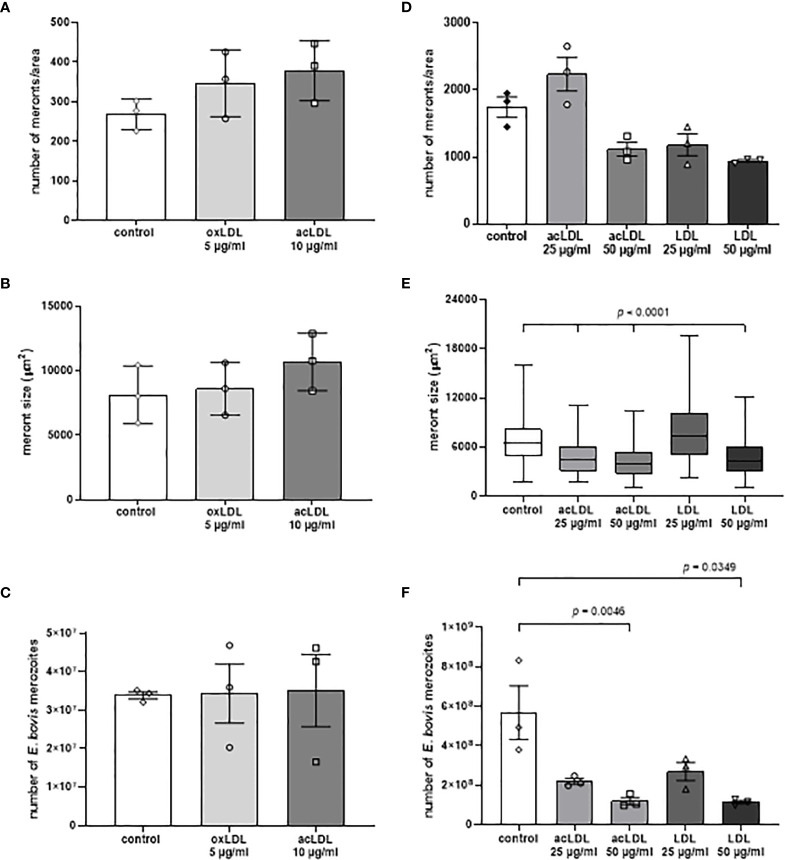
Supplementation of LDL, aLDL and oxLDL failed to boost parasite replication. The effects of LDL, acLDL and oxLDL supplementation on E. bovis development were studied by estimating meront I numbers **(A, D)** and sizes **(B, E)** in addition to merozoite I numbers **(C, F)**. All treatments failed to significantly boost parasite replication. Three biological replicates; significance p < 0.05. Mean and standard deviation.

To estimate whether different LDL types would affect macromeront development, non-modified LDL, oxLDL, and acLDL were continuously supplemented to *E. bovis*-infected cell cultures from 10 d p.i. onwards. Overall, after supplementation with oxLDL and acLDL at lower concentrations (5 and 10 µg/ml, respectively), the number of meronts per area (**Figure 4A**) and meronts size (**Figure 4B**) increased by tendency, but no differences in merozoite I production were observed when compared with the plain medium (**Figure 4C**). Higher LDL concentrations were only applied in cases of acLDL and LDL, since these revealed to be toxic in case of oxLDL. However, even though meront I numbers (per area of infected cell layer) seemed increased by tendency after supplementation with 25 µg/ml acLDL, meronts I revealed smaller than in controls and produced less merozoites I (**Figures 4D–F**). Higher acLDL and LDL concentrations even proved detrimental for parasite replication and led to significantly smaller macromeronts (**Figure 4E**) and to reduced merozoite I production when compared with control cultures (**Figure 4F**).

### LOX-1 Expression Is Enhanced During *Eimeria bovis* Merogony I in BUVEC and Its Soluble Form Is Decreased in Blood Samples of Infected Calves

**Figure 5:**
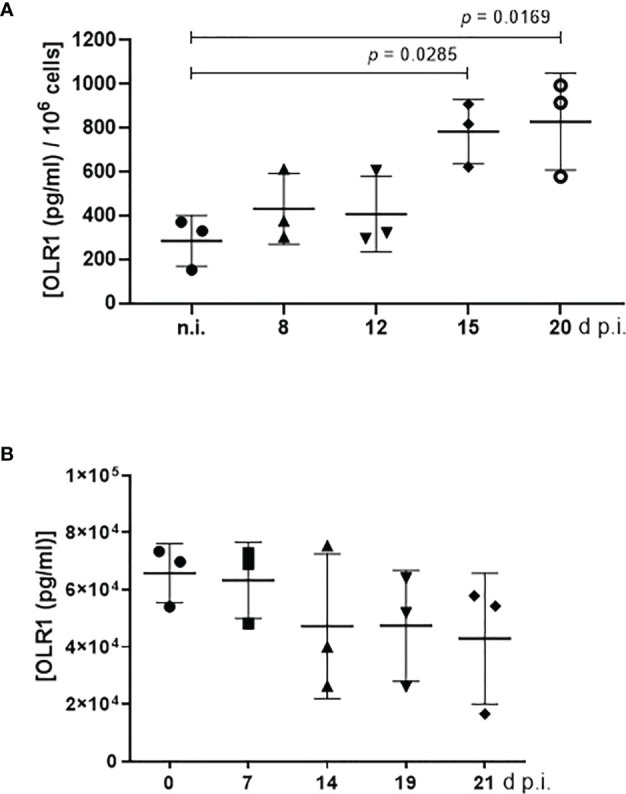
LOX-1 (syn. OLR1) abundance in *E. bovis*-infected bovine umbilical vein endothelial cells (BUVEC) and in blood samples of *E. bovis*-infected calves. **(A)** LOX-1 abundance in *E. bovis*-infected BUVEC was monitored during first merogony and revealed an increase in protein concentration [(pg/ml)/10^6^ cells] over time (*n* = 3, technical replicates). **(B)** Blood samples of three experimentally infected calves were examined for soluble LOX-1 levels throughout infection. Soluble LOX-1 was not significantly altered during infection. Mean and standard deviation; significance *p <*0.05.

LOX-1 is documented to support both oxLDL and acLDL internalization (Mango et al., 2011). Here, LOX-1 expression was quantified *via* a commercial ELISA in *E. bovis*-infected host cells (infection rates: 23.40% ± 5.81%) at 8, 12, 15, and 20 d p.i. and compared with parallel processed non-infected controls (**Figure 5A**). Considering total merogony I, LOX-1 concentration increased over time and especially toward the end of macromeront formation. At 8 and 12 d p.i., i.e., at immature meront status, no differences in LOX-1 expression were apparent. With ongoing macromeront maturation at 15 and 20 d p.i., a significant increase in LOX-1 expression was registered (**Figure 5A**). Given that LOX-1 is significantly involved in both acLDL and oxLDL incorporation, these data may reflect an increased demand of *E. bovis*-infected cells for LDL types delivering cholesterol for successful replication.

LOX-1 is mainly expressed on the cell surface of endothelial cells, macrophages, smooth muscle cells, and platelets (Mango et al., 2011), but may additionally be cleaved at its membrane proximal extracellular domain and released as soluble form into the blood *in vivo* (Hayashida et al., 2005). Based on current *in vitro* findings and to monitor the concentration of soluble LOX-1 molecules *in vivo*, blood samples of experimentally *E. bovis*-infected calves (*n* = 3) were tested for this molecule during the course of prepatency and patency. As a common observation of non-syngeneic animals, individual reactions varied considerably thereby hampering significant differences throughout the study period. Thus, basal levels of plasmatic LOX-1 were similar in all animals at the day of infection (0 d p.i.), but then—as a tendency—seemed to decrease with ongoing infection (**Figure 5B**). Lower concentrations were especially detected during patency (19 and 21 d p.i.). Of note, infected calves shed oocysts from days 18 to 23 p.i. [peaking at 19–20 days p.i. with a mean of 72,766 and 17,850 oocysts per gram of feces (OPG), respectively], i.e., as reported before, parasite development occurs faster *in vivo* and first merogony is usually completed from days 14–15 p.i. onwards. The observation of inverse LOX-1 abundance in blood samples and endothelial cells represents an interesting finding since it may indicate that even with an *E. bovis*-driven upregulation of LOX-1 on endothelial cells, less soluble molecules are present in the serum, i.e., LOX-1 remains locally where the intracellular parasite needs it to guarantee boosted lipid uptake.

### *Eimeria bovis* Merozoite I Replication Is Blocked by Sterol Uptake Inhibition

**Figure 6:**
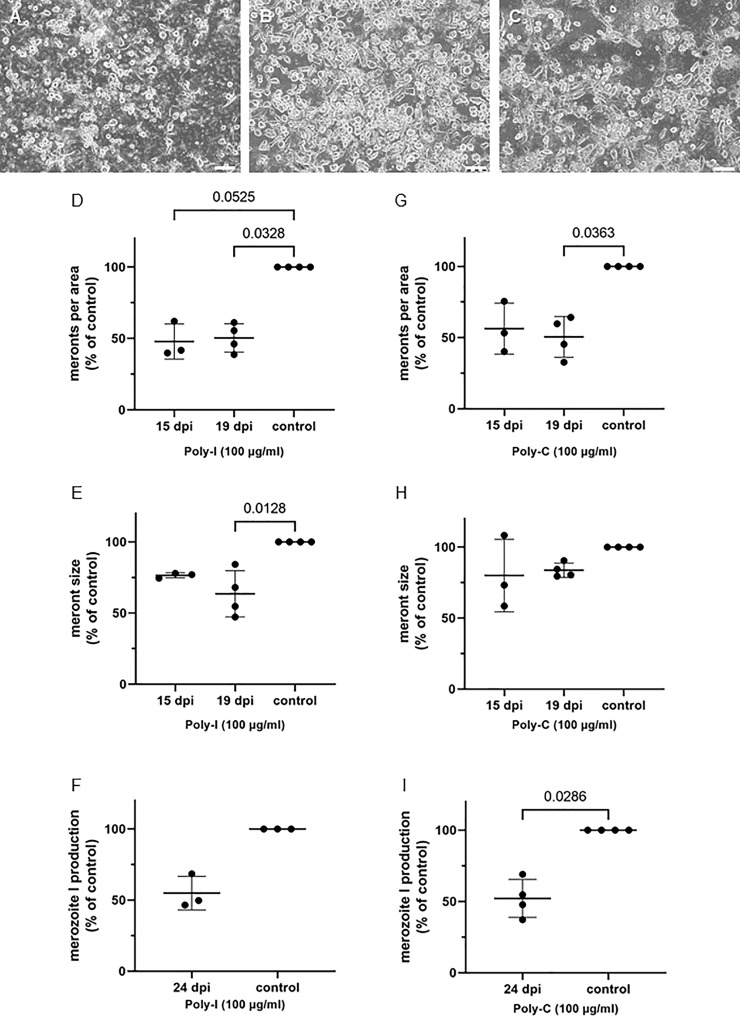
Poly-I (polyinosinic acid) and Poly-C (polycytidic acid) treatments reduce E. bovis replication. **(A)** Poly-I treated-, **(B)** control- and **(C)** Poly-C treated- *E. bovis*-infected BUVEC (3-4 biological replicates with technical duplicates) at day 19 p. i. Number of meronts I **(D, G)** and meront I size **(E, H)** were calculated at 15 and 19 d p. i., and merozoite I production **(F, I)** was determined at 24 d p. i. Results are expressed as percentage of untreated controls (100%). Scale bar 200 µm.

**Figure 7:**
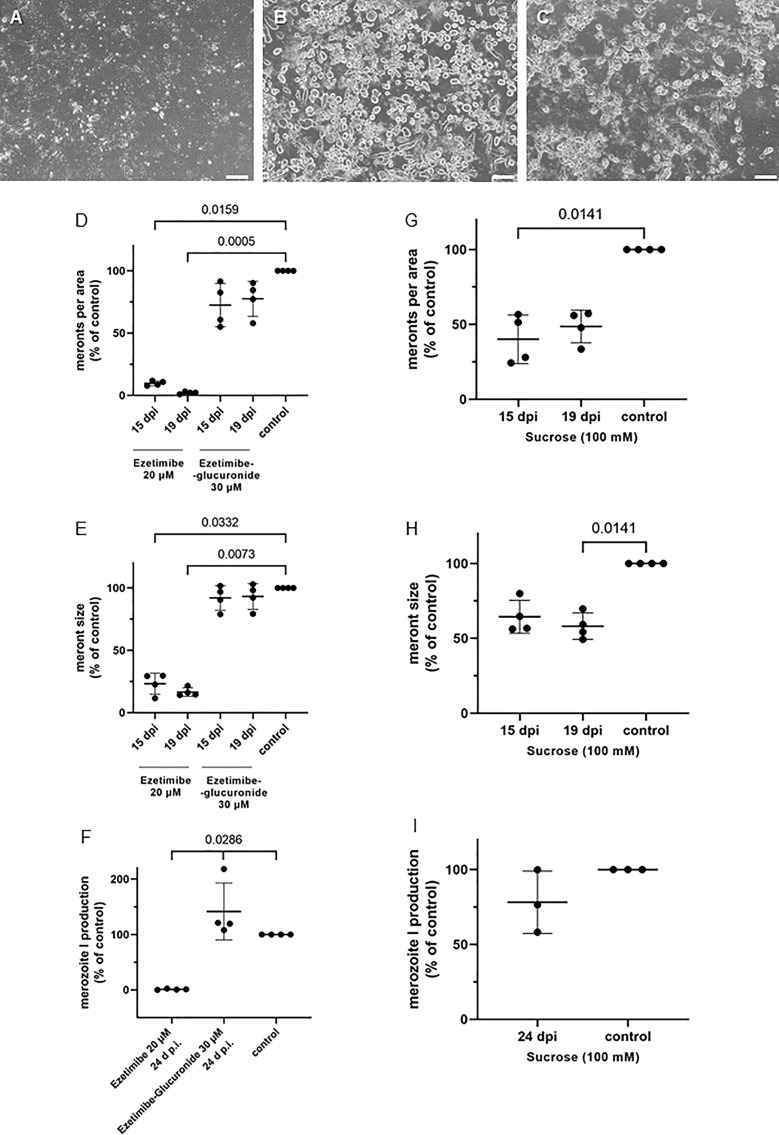
Effects of ezetimibe, ezetimibe-glucuronide and sucrose treatments on E. bovis development. **(A)** ezetimibe treated-, **(B)** control- and **(C)** sucrose treated- *E. bovis*-infected BUVEC (3-4 biological replicates with technical duplicates) at 19 d p. i. Number of meronts I **(D, G)** and meront I size **(E, H)** were calculated at 15 and 19 d p. i., and merozoite I production **(F, I)** was determined at 24 d p. i. Results are expressed as percentage of untreated controls (100%). Scale bar 200 µm.

To define the significance of host cellular lipid supplementation for *E. bovis* macromeront formation, the effects of different inhibitors of sterol uptake were analyzed at times of progressive macromeront growth and maturation, i.e., at days 15 (immature meront), 19 (mature meront), and 24 (merozoite I release) p.i. As read-out, the number and the size of macromeronts as well as merozoite I production were determined. For inhibitor studies, poly-I and poly-C (**Figure 6**), as well as ezetimibe, ezetimibe-glucuronide, and sucrose (**Figure 7**), were tested.

Poly-I, a four-stranded polyribonucleotide, is an effective competitor of scavenger receptors (Krieger and Herz, 1994), especially of scavenger receptor A (Liu et al., 2012). On the other hand, poly-C, a structural homologous of poly-I, is also a ligand but has no reported scavenger receptor binding activity (Dinguirard and Yoshino, 2006). Overall, poly-I and poly-C inhibited parasite proliferation at different levels (**Figure 6**). While poly-I significantly decreased the numbers of meronts at days 15 and 19 p.i. at about 50% of controls (*p* = 0.0525 and *p* = 0.0328, respectively; **Figures 6A, D**), the poly-C treatment-induced decline was only significant at day 19 p.i. (*p* = 0.0363) with an overall reduction of approximately 50% of controls (**Figures 6A, G**). However, the meront size was not significantly reduced with poly-C treatments at any time, while poly-I treatments led to the formation of smaller meronts at 19 d p.i. (*p* = 0.0128) (**Figures 6E, H**). Nevertheless, parasite replication was reduced by 50% at 24 d p.i. with both treatments, i.e., poly-C (*p* = 0.0286) and poly-I (not significant) (**Figures 6F, I**).

More prominent effects were observed with ezetimibe treatments (**Figure 7**). Ezetimibe is a well-known hypolipidemic drug capable of reducing intestinal cholesterol absorption *via* blockage of the Niemann–Pick C-1 like-1 protein (NPC1L1) endocytosis into clathrin-coated vesicles and thereby diminishing cholesterol internalization into enterocytes (Ge et al., 2008; Wang et al., 2009). Moreover, the class B type 1 scavenger receptor (SR-BI) and the aminopeptidase N (CD13) were additionally reported as potential ezetimibe targets (Kramer et al., 2005; Labonté et al., 2007). Of note, *E. bovis* macromeront development was almost completely blocked by ezetimibe treatments (**Figures 7A, D–F**), especially at days 19 and 24 p.i. (*p* < 0.05). It has to be mentioned that treatments started after early immature meronts had already formed (at 10 d p.i.). Ezetimibe treatments totally abrogated any further development or maturation of meronts I. Even though a strong reduction in the number of developing macromeronts was stated (**Figures 7A, D**; 15 d p.i.: *p* = 0.0159; 19 d p.i.: *p* = 0.0005), few meronts I were still detectable but were more than 75% smaller than those in controls (**Figure 7E**; *p* = 0.0332 and *p* = 0.0073, at 15 and 19 d p.i., respectively). Moreover, merozoite I production was barely detectable after ezetimibe treatments at 24 d p.i. (**Figure 7F**, *p* = 0.0286). Noteworthy, treatments with ezetimibe-glucuronide, the most important pharmacologically active metabolite of ezetimibe following *in vivo* liver biotransformation, had no significant effects on *E. bovis* proliferation (**Figures 7D–F**).

Less prominent effects were induced by sucrose (100 mM), a non-specific inhibitor of LDL internalization. Nevertheless, sucrose treatments reduced meront I numbers (15 d p.i.: *p* = 0.0141; 19 d p.i.: *p* = n.s.) and sizes (15 d p.i.: *p* = n.s.; 19 d p.i.: *p* = 0.0141) by approximately 50% (**Figures 7G–I**). However, parasite replication was not significantly affected by sucrose treatments, nevertheless inducing a 25% reduction in merozoite I production (**Figure 7I**).

### Inhibitors of Intracellular Sterol Trafficking and Release From Endosomes Block *Eimeria bovis* Development and Merozoite I Proliferation

**Figure 8:**
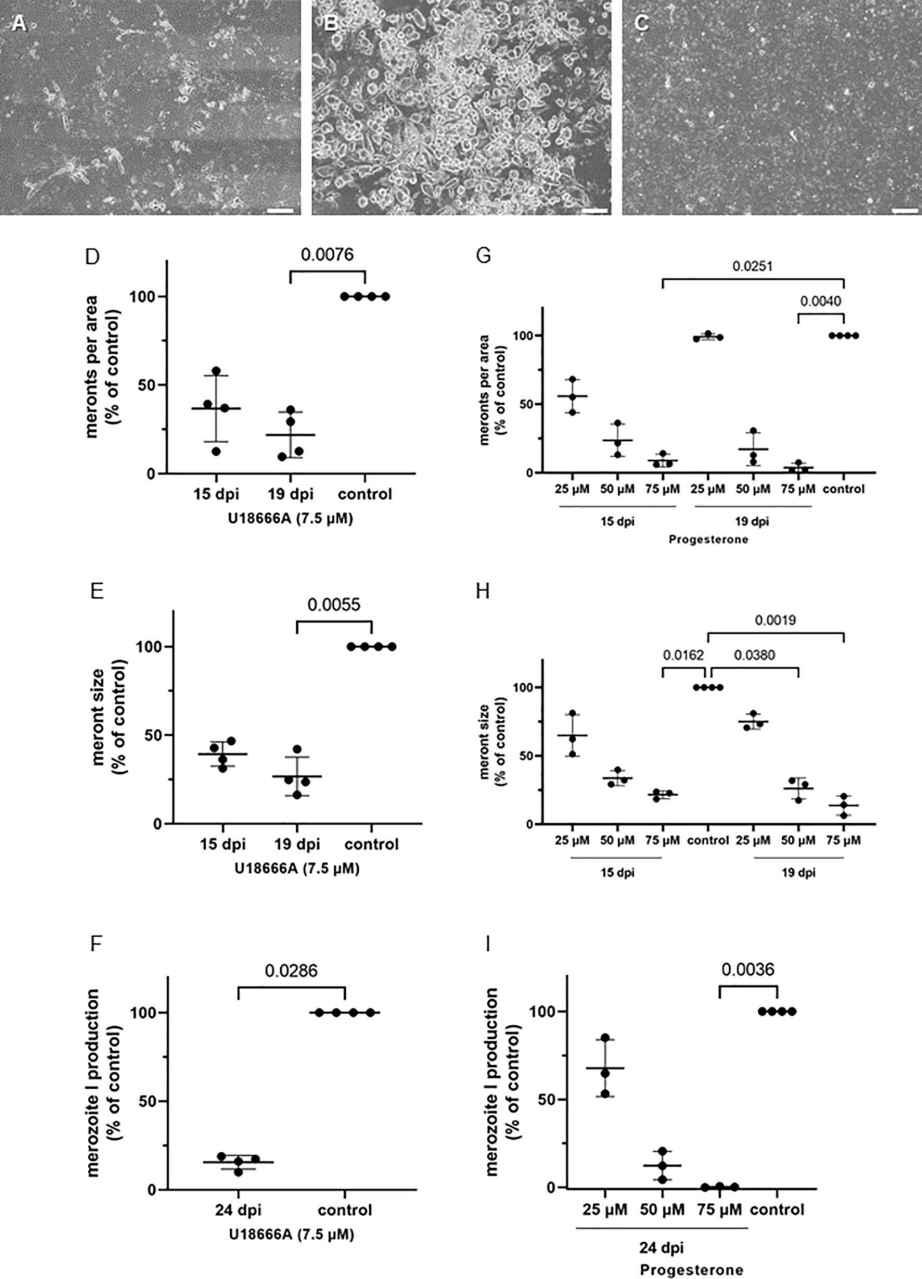
U18666A and progesterone treatments block E. bovis replication. **(A)** U18666A treated-, **(B)** control- and **(C)** progesterone treated- E. bovis-infected BUVEC (3-4 biological replicates with technical duplicates) at 19 d p. i. Number of meronts I **(D, G)** and meront I size **(E, H)** were calculated at 15 and 19 d p. i., and merozoite I production **(F, I)** was determined at 24 d p. i. Results are expressed as percentage of untreated controls (100%). Scale bar 200 µm.

The blockage of cholesterol trafficking from late endosomes/lysosomes *via* progesterone and U18666A treatments was studied at days 15 and 19 p.i. (**Figure 8**). These substances cause an accumulation of cholesterol in late endosomes/lysosomes, leaving this molecule unavailable for parasite exploitation. Overall, treatments with U18666A (7.5 µM), which can directly bind to the sterol-sensing domain of the NPC1 (Niemann–Pick 1) lysosomal membrane protein (Lu et al., 2015), induced a significant blockage of *E. bovis* macromeront development at 19 d p.i. (**Figures 8A, D–F**). Thus, the number of meronts I per area was significantly lower (*p* = 0.0076) in treated cultures when compared with controls at 19 d p.i. (reduction of 75%; **Figures 8A, D**) with these effects being slightly less prominent at 15 d p.i. with 60% less meronts I per area (**Figure 8D**). Those meronts still present in treated cultures showed significant smaller sizes (19 d p.i.: *p* = 0.0055; 15 d p.i.: *p* = not significant) (**Figure 8E**). In addition, U18666A treatments resulted in a significant inhibition (*p* = 0.0286) of *E. bovis* replication leading to a reduction of 75% in merozoite I production (**Figure 8F**).

Progesterone has been described as a blocker of cholesterol translocation from lysosomes (Butler et al., 1992). Both progesterone and U18666A may block LDL-cholesterol transfer by inhibiting NPC1 shuttling (Liscum, 2000). Here, the inhibitory effects of progesterone treatments showed a dose dependency (**Figures 8G–I**). Treatments of *E. bovis* cultures at 75 µM progesterone resulted in an almost entire blockage of merogony I with only few meronts I being able to proceed further development (**Figures 8C, G–I**). Thus, the number of meronts I was reduced for almost 90% at 15 and 19 d p.i. (*p* = 0.0251 and *p* = 0.0040, respectively) and meront I sizes were significantly diminished (75 µM: 15 d p.i.: *p* = 0.0162, 19 d p.i.: *p* = 0.0019; 50 µM: 19 d p.i.: *p* = 0.0380). Likewise, offspring production was dramatically reduced by progesterone treatments at both 50 and 75 µM leading to almost complete blockage of parasite replication at 75 µM treatments (**Figure 8I**, *p* = 0.0036).

## Discussion

Coccidian parasites are considered defective for *de-novo* cholesterol biosynthesis and, as such, must scavenge cholesterol from their infected host cells by either enhancing uptake of cholesterol from extracellular sources or by upregulating host cell *de-novo* biosynthesis (Bansal et al., 2005; Labaied et al., 2011; Coppens, 2013; Ehrenman et al., 2013; Hamid et al., 2014). Since the formation of huge parasitic stages, such as *E. bovis* macromeronts (>300 µm in size), is a highly cholesterol-demanding process, we here examined the role of both host cellular uptake of different LDL variations and intracellular cholesterol trafficking during *E. bovis* first merogony in endothelial cells.

Lipid droplets are key storage organelles of cellular lipid and energy homeostasis. They are involved in several cellular functions, such as lipid and energy metabolism, but also in membrane trafficking and cell signaling (Vallochi et al., 2018). Moreover, these organelles were reported of importance for parasitic infections (Palacpac et al., 2004; Hamid et al., 2015; Vallochi et al., 2018; Silva et al., 2019). Previous analyses on lipid droplets distribution in *E. bovis* macromeronts showed a considerable enhancement in numbers, but exclusively for fixed host cells (Hamid et al., 2015). In contrast, novel live-cell holotomography uses fixation- and label-free 3D techniques (Sandoz et al., 2018; Sandoz et al., 2019). In the current work, the latter methodology confirmed an enhanced presence of lipid droplet-like structures in *E. bovis*-infected BUVEC from 8 d p.i. onwards. The fact that these structures were also stained by LipidSpot™ proved them as classical lipid droplets. To control the precise position of lipid droplets in the complex of *E. bovis*-macromeront-carrying BUVEC, LipidSpot™-based staining was also applied for confocal microscopy and confirmed intrameront positions of lipid droplets from day 12 p.i. onwards. Interestingly, the number and distribution of lipid droplets depended on macromeront maturation. Hence, different phenotypes of intrameront lipid droplet positioning were observed, since in immature meronts I, these organelles seemed organized in few but large and rather centrally positioned clusters while they were rather evenly dispersed in mature macromeronts showing merozoite I formation. So far, the nature of these peculiar lipid droplet accumulations in immature *E. bovis* meronts is unknown. Given that lipid droplets are reported to associate with different cellular organelles, such as lysosomes, mitochondria, or peroxisomes (Olzmann and Carvalho, 2019), these clusters may also be linked to certain schizogony-related structures. The fact that lipid droplets seemed larger at early merogony than at mature meront stage may reflect typical lipid droplet dynamics comprising lipid droplet shrinkage and expansion due to lipid droplet biogenesis, fusion, and degradation driven by cellular lipid needs (Olzmann and Carvalho, 2019). It appears likely that smaller but numerous lipid droplets observed at the end of first merogony may be linked to an enhanced parasite-driven lipid consumption needed for numerous offspring formation (>170,000 merozoites I). In the case of *T. gondii* infections, Nolan et al. (2017) described host lipid droplets as an important source of lipids for these intracellular parasites. Bodipy 493/503-marked lipid droplets were observed not only in the cytoplasm of host cells but also within the PV. Likewise, Gomes et al. (2014) speculated that lipid droplets are recruited to the PV in *T. gondii*-infected skeletal muscle cells, and Mota et al. (2014) suggested the same in infected macrophages. In contrast to current findings, in *B. besnoiti*-infected BUVEC, lipid droplets were mostly observed in the host cell cytoplasm but not within meronts (Silva et al., 2019). Nevertheless, it remains unclear if lipid droplets are formed by the parasite itself either based on their capacity to associate with other cellular organelles *via* membrane contact sites (Olzmann and Carvalho, 2019) or internalized from the host cell compartment as previously hypothesized (Hamid, 2014). However, these differences in lipid droplet positioning might reflect the different needs of lipids by fast replicating parasites (e.g., *B. besnoiti*, *T. gondii*) for immediate offspring formation, contrasting with continuous high demand over time in the slow replicating parasite *E. bovis*.

Exogenous sterol uptake *via* LDL constitutes an important mechanism of cholesterol delivery since LDL classically is enriched in free cholesterol and cholesteryl esters (Gerl et al., 2018). Besides non-modified LDL, acLDL and oxLDL are also internalized by endothelial cells (Voyta et al., 1984; Valente et al., 2014), and by being present in blood or lymph, all LDL modifications in principle contribute to cholesterol supply *in vivo*. Likewise, an internalization of all three LDL types into BUVEC was here observed. However, infection-driven differences in cellular LDL distribution were apparent since non-modified LDL and oxLDL (to a less degree) were found in both the host cell cytoplasm and macromeronts, while acLDL failed to reach the macromeronts. These data confirm that *E. bovis* is in principle capable to import and use LDL (Hamid et al., 2015) and, to a less extent, oxLDL, but fails to use acLDL as an exogenous cholesterol source. The principal capacity to utilize LDL for obligate intracellular replication is in line with previous reports on other apicomplexan parasites, such as *T. gondii*, *C. parvum*, or *Plasmodium* spp. (Coppens et al., 2000; Labaied et al., 2011; Ehrenman et al., 2013; Ihara and Nishikawa, 2014). However, acLDL and oxLDL supplementation at 5–10 µg/ml had no impact on *E. bovis* merozoite I generation. In contrast, LDL supplementation was previously shown to boost the proliferation of *E. bovis*, *T. gondii* (in CHO cells), or *B. besnoiti* (Coppens et al., 2000; Hamid et al., 2015; Silva et al., 2019). Furthermore, higher acLDL and LDL concentrations even induced a decrease in *E. bovis* replication, which may reflect the well-known toxic effects of lipids at deregulated cellular concentrations. In accordance to the above findings, LDL supplementation failed to affect the replication of *Plasmodium yoelli*, *Plasmodium berghei* (Labaied et al., 2011), *C. parvum* (Ehrenman et al., 2013), and *T. gondii* in macrophages (Nishikawa et al., 2011), indicating that these reactions are species- and host cell type-dependent. Even though parasites require high amounts of cholesterol for successful replication, they face the challenge of preventing intracellular imbalances, which will impair host cell metabolism and consequently hamper parasite proliferation (Taubert et al., 2018), as observed here. Especially in case of oxLDL, adverse effects are well documented and include endothelial cell death by activation of NF-κB and activator protein-1 (AP-1) pathways (Valente et al., 2014) or endothelial dysfunction alongside ROS generation and nitric oxide (NO) synthesis inhibition (Mango et al., 2011). In line with this, oxLDL >5 µg/ml here revealed to be cytotoxic for primary BUVEC layers.

LOX-1 is an important receptor of oxLDL uptake in endothelial cells (Mango et al., 2011; Akhmedov et al., 2014; Barreto et al., 2021) but is also able to bind acLDL at comparable affinity (Kumano-Kuramochi et al., 2012). Other oxLDL-related known receptors are, e.g., the scavenger receptor CD36, the scavenger receptor class A member 1 (SCARA1), and the G-protein-coupled receptor angiotensin II type 1 receptor (AT1) (Silverstein, 2018; Pandey et al., 2020; Cheng et al., 2021; Takahashi et al., 2021). Previously, *E. bovis*-infected host cells have been shown to upregulate LOX-1 gene transcription during first merogony (Taubert et al., 2010; Hamid et al., 2015). Here, we confirmed an *E. bovis*-driven upregulation of LOX-1 protein expression toward the end of macromeront formation (days 15 and 20 p.i.) *in vitro* thereby indirectly confirming the findings on oxLDL internalization. Likewise, parasite-triggered LOX-1 induction was also recently reported for *B. besnoiti*-infected host endothelial cells (Silva et al., 2019) and *T. gondii*- (Masek, 2006) and *P. chabaudi*-infected cells (Marrelli et al., 2020), thereby potentially indicating a more general mechanism of apicomplexans. We additionally tested the blood samples of experimentally infected calves for LOX-1 content. Interestingly, we found an inverse relationship with reactions of host cells since decreasing levels of LOX-1 were present in blood samples with ongoing merogony I. As such, an infection-driven influence on the mode of LOX-1 presentation may be hypothesized. Given that the parasite would only profit from membrane-bound LOX-1 leading to improved host cellular LDL uptake, an inhibition of LOX-1 release leading to lower plasmatic LOX-1 concentrations seems plausible. However, further future detailed studies are needed to verify this assumption.

LDL-mediated endocytosis represents the main uptake mechanism of cholesterol in many cell types (Simons and Ikonen, 2000), and several parasites were reported to critically rely on this mechanism for successful replication (Coppens et al., 2000; Hamid et al., 2015; Nolan et al., 2015; Silva et al., 2019). To test the importance of different steps of lipid internalization for *E. bovis*, we here applied different inhibitors targeting lipid uptake and transport. First, we used the scavenger receptor ligand poly-I (Xu et al., 2008; Liu et al., 2012), which is a four-stranded polyribonucleotide and an effective competitor of scavenger receptors (Krieger and Herz, 1994), especially of scavenger receptor A (Liu et al., 2012). We also tested poly-C, a structural homologous of poly-I. This substance is a ligand but has no reported scavenger receptor binding activity to scavenger receptors (Dinguirard and Yoshino, 2006). Overall, both treatments in principle diminished *E. bovis* macromeront formation and merozoite I production, even though with moderate effects. In line with this, in the case of *Schistosoma mansoni* sporocysts, poly-I inhibited acLDL binding to sporocyst tegument; however, poly-C exhibited little inhibition on acLDL binding activity (Dinguirard and Yoshino, 2006).

We furthermore used ezetimibe, which is a well-known blocker of NPC1L1-mediated micellar cholesterol absorption in the intestine (Davis et al., 2004; Garcia-Calvo et al., 2005) and is clinically applied as a cholesterol-lowering drug. This drug moderately inhibited *C. parvum* proliferation *in vitro* (Ehrenman et al., 2013) and diminished *Leishmania* infection *in vivo* (Andrade-Neto et al., 2016). Overall, ezetimibe treatments of *E. bovis*-infected BUVEC led to a drastic inhibition of macromeront development and merozoite I production. However, when we applied ezetimibe-glucuronide, which is reported as biologically active metabolite of ezetimibe *in vivo* (Garcia-Calvo et al., 2005), this compound entirely failed to affect parasite development thereby suggesting NPC1L1-independent antiparasitic ezetimibe effects. These findings are in line with recent studies of Larrazabal et al. (2021) who showed that ezetimibe but not ezetimibe-glucuronide effectively blocked *B. besnoiti*, *N. caninum*, and *T. gondii* infections in BUVEC. Noteworthy, NPC1L1 seems hardly transcribed in BUVEC and may therefore not represent the true drug target in this cell type (Larrazabal et al., 2021). Of note, besides its effects on NPC1L1, ezetimibe also seems to act as an ACAT2 (acetyl-CoA acetyltransferase 2) inhibitor (Clader, 2004). Interestingly, both ACAT1 and ACAT2 gene transcripts were significantly upregulated in *E. bovis*-infected BUVEC (Hamid et al., 2015). ACAT1 and ACAT2 are involved in the formation of acetoacetyl-CoA, which represents an important substrate in the mevalonate pathway (Goldstein et al., 2006; Hamid et al., 2015), corroborating the involvement of cholesterol *de-novo* biosynthesis in *E. bovis* replication (Hamid et al., 2015). Moreover, Orsó et al. (2019) reported that ezetimibe but not ezetimibe-glucuronide reduced the cellular content in cholesteryl esters in an NPC1L1-independent manner in human monocytes. Given that the presence of cholesteryl esters was recently proven as essential for *E. bovis* proliferation (Hamid, 2014), this mechanism may also have contributed to current ezetimibe-driven antiparasitic effects.

Sucrose is a non-specific blocker of receptor-mediated endocytosis (Heuser and Anderson, 1989; Nieland et al., 2005) and an inhibitor of lysosomal function (Park et al., 1988; Coppens et al., 2000). In the current work, sucrose treatments blocked *E. bovis* macromeront formation but failed to reduce significantly merozoite I production. In line with current findings, sucrose treatments inhibited cholesteryl oleate and cholesterol transport in *T. gondii*-infected host cells (Coppens et al., 2000). So far, it remains unclear if one of the above-stated mechanisms mediated sucrose-related effects on *E. bovis*. It seems plausible that both mechanisms contributed to these effects since both functional LDLR (Hamid et al., 2014; Hamid et al., 2015) and proper lysosomal function are pivotal for *E. bovis* replication. Referring to the latter, the current data showed that inhibitors of lysosomal function, such as U18666A and progesterone, effectively inhibited parasite macromeront formation thereby emphasizing the pivotal role of cholesterol/lipid modification and transport for effective parasite proliferation. Endocytosed lipoproteins are delivered to endosomes and their protein/phospholipid coat is degraded in late endosomes/lysosomes, yielding free cholesteryl esters that will be hydrolyzed to cholesterol (Coppens et al., 2000). Both U18666A and progesterone block cholesterol translocation from late endosomes/lysosomes, sequestering cholesterol inside these organelles (Liscum and Faust, 1989; Coppens et al., 2000; Cenedella, 2009; Lu et al., 2015; Petersen et al., 2017). In line with our findings, progesterone and U18666A treatments also caused cumulative effects on *T. gondii* leading to a remarkable reduction of cholesterol associated with the parasites and a consequent slower replication (Coppens et al., 2000).

In summary, current data emphasize that *E. bovis*-driven modulation of host endothelial cell functions seems crucial to satisfy its extraordinary demands for cholesterol during massive asexual intracellular replication. The relevance of exogenous sterol uptake and of intracellular cholesterol trafficking for successful massive parasite proliferation unveils new perspectives for the development of novel drug targets not only against *E. bovis* and closely related pathogenic ruminant *Eimeria* but also against other apicomplexan species infecting humans, wildlife, and domestic animals.

## Data Availability Statement

The original contributions presented in the study are included in the article/**Supplementary Material**. Further inquiries can be directed to the corresponding author.

## Ethics Statement

All animal procedures were performed according to the Justus Liebig University (JLU) Giessen Animal Care Committee guidelines, approved by the Ethic Commission for Experimental Animal Studies of the State of Hesse (Regierungspräsidium Giessen), and were in accordance with the current German Animal Protection Laws and European animal welfare legislation: ART13TFEU. Identification number of animal care license: GI 18/10-Nr. A2/2016, JLU 589_AZ (*Eimeria bovis* oocyst production).

## Author Contributions

LS, ZV, CH, and AT conceived and designed the experiments. LS, ZV, SL-O, and AT performed the experiments. All authors performed the analyses and interpretation of the data. LS, SL-O, and AT prepared the manuscript. LS and ZV prepared the figures. LS performed the statistical analyses. All authors revised and approved the final version of the manuscript.

## Funding

Selected experiments were supported and financed by the German Research Foundation [Deutsche Forschungsgemeinschaft (DFG); grant number: TA 291/10-1]. The publication fees were partially funded by the Open Access Funds of Justus Liebig University Giessen.

## Conflict of Interest

The authors declare that the research was conducted in the absence of any commercial or financial relationships that could be construed as a potential conflict of interest.

## Publisher’s Note

All claims expressed in this article are solely those of the authors and do not necessarily represent those of their affiliated organizations, or those of the publisher, the editors and the reviewers. Any product that may be evaluated in this article, or claim that may be made by its manufacturer, is not guaranteed or endorsed by the publisher.

## Supplementary Material

Supplementary Figure 1 Survival assays.

Supplementary Figure 2 Z-stack oxLDL confocal.

Supplementary Figure 3 acLDL z-stacks confocal.

Supplementary Video 1 Video of 3D reconstruction of confocal analysis of *E. bovis* immature macromeront carrying lipid droplets at 15 d p. i.

Supplementary Video 2 Video of 3D reconstruction of confocal analysis of *E. bovis* mature macromeront with several smaller lipid droplets at 22 d p. i.
