# Supplementary figures and images for: Novel Insights Into Sterol Uptake and Intracellular Cholesterol Trafficking During Eimeria bovis Macromeront Formation
Source: Front Cell Infect Microbiol. 2022 Feb 11;12:809606. doi: 10.3389/fcimb.2022.809606 (PMC8878908; doi:10.3389/fcimb.2022.809606)

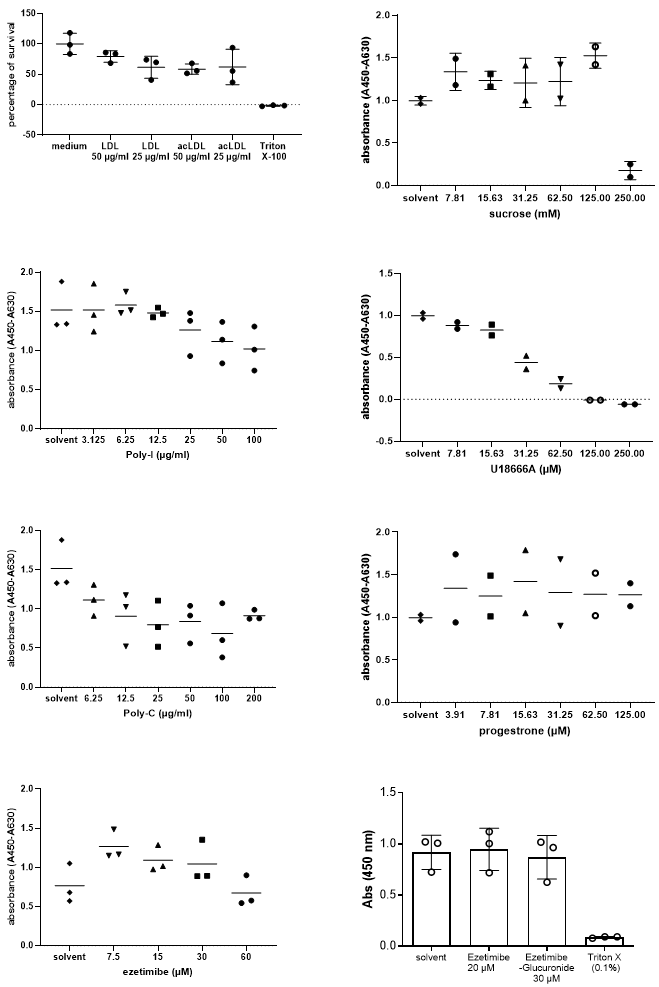

Supplement: Supplementary Figure 1 — Survival assays. [file Image_1.tif]

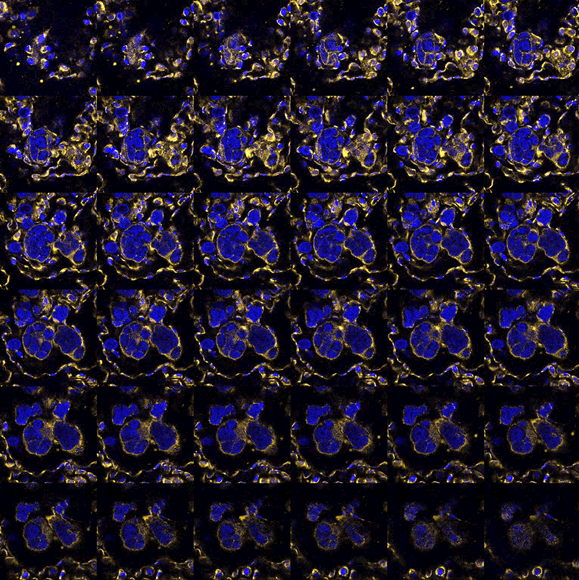

Supplement: Supplementary Figure 2 — Z-stack oxLDL confocal. [file Image_2.tif]

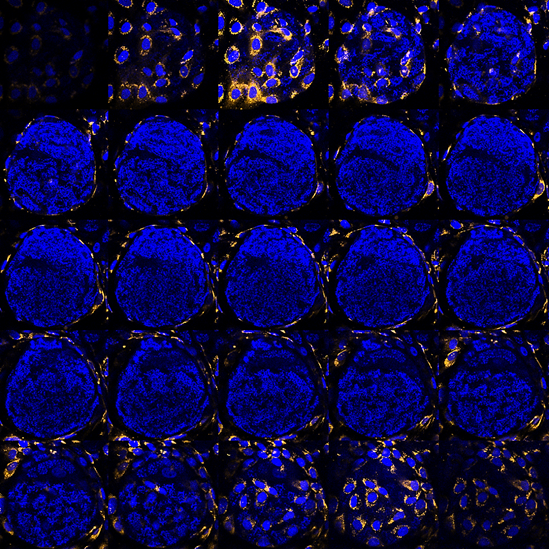

Supplement: Supplementary Figure 3 — acLDL z-stacks confocal. [file Image_3.tif]
